# Extracellular vesicles and pasteurized cells derived from *Akkermansia muciniphila* protect against high-fat induced obesity in mice

**DOI:** 10.1186/s12934-021-01709-w

**Published:** 2021-12-04

**Authors:** Fatemeh Ashrafian, Shahrbanoo Keshavarz Azizi Raftar, Arezou Lari, Arefeh Shahryari, Sara Abdollahiyan, Hamid Reza Moradi, Morteza Masoumi, Mehdi Davari, Shohreh khatami, Mir Davood Omrani, Farzam Vaziri, Andrea Masotti, Seyed Davar Siadat

**Affiliations:** 1 Microbiology Research Center (MRC), Pasteur Institute of Iran, Tehran, Iran; 2 Clinical Research Department, Pasteur Institute of Iran, Tehran, Iran; 3 Systems Biomedicine Unit, Pasteur Institute of Iran, Tehran, Iran; 4 Basic and Molecular Epidemiology of Gastrointestinal Disorders Research Center, Research Institute for Gastroenterology and Liver Diseases, Shahid Beheshti University of Medical Sciences, Tehran, Iran; 5 Department of Basic Sciences, School of Veterinary Medicine, Shiraz University, Shiraz, Iran; 6 Department of Mycobacteriology and Pulmonary Research, Pasteur Institute of Iran, Tehran, Iran; 7 Department of Biochemistry, Pasteur Institute of Iran, Tehran, Iran; 8 Department of Medical Genetics, Faculty of Medicine, Shahid Beheshti University of Medical Sciences, Tehran, Iran; 9 Research Laboratories, Children’s Hospital Bambino Gesù-IRCCS, Rome, Italy

**Keywords:** *Akkermansia muciniphila*, Extracellular vesicles, Obesity, Pasteurization

## Abstract

**Background:**

Several studies have shown that probiotics have beneficial effects on weight control and metabolic health. In addition to probiotics, recent studies have investigated the effects of paraprobiotics and postbiotics. Therefore, we evaluated the preventive effects of live and pasteurized *Akkermansia muciniphila* MucT (*A. muciniphila*) and its extracellular vesicles (EVs) on HFD-induced obesity.

**Results:**

The results showed that body weight, metabolic tissues weight, food consumption, and plasma metabolic parameters were increased in the HFD group, whereas *A. muciniphila* preventive treatments inhibited these HFD. The effects of pasteurized *A. muciniphila* and its extracellular vesicles were more noticeable than its active form. The HFD led to an increase in the colonic, adipose tissue, and liver inflammations and increased the expression of genes involved in lipid metabolism and homeostasis. Nevertheless, these effects were inhibited in mice that were administered *A. muciniphila* and its EVs. The assessment of the gut microbiota revealed significant differences in the microbiota composition after feeding with HFD. However, all treatments restored the alterations in some bacterial genera and closely resemble the control group. Also, the correlation analysis indicated that some gut microbiota might be associated with obesity-related indices.

**Conclusions:**

Pasteurized *A. muciniphila* and its EVs, as paraprobiotic and postbiotic agents, were found to play a key role in the regulation of metabolic functions to prevent obesity, probably by affecting the gut-adipose-liver axis.

**Graphical Abstract:**

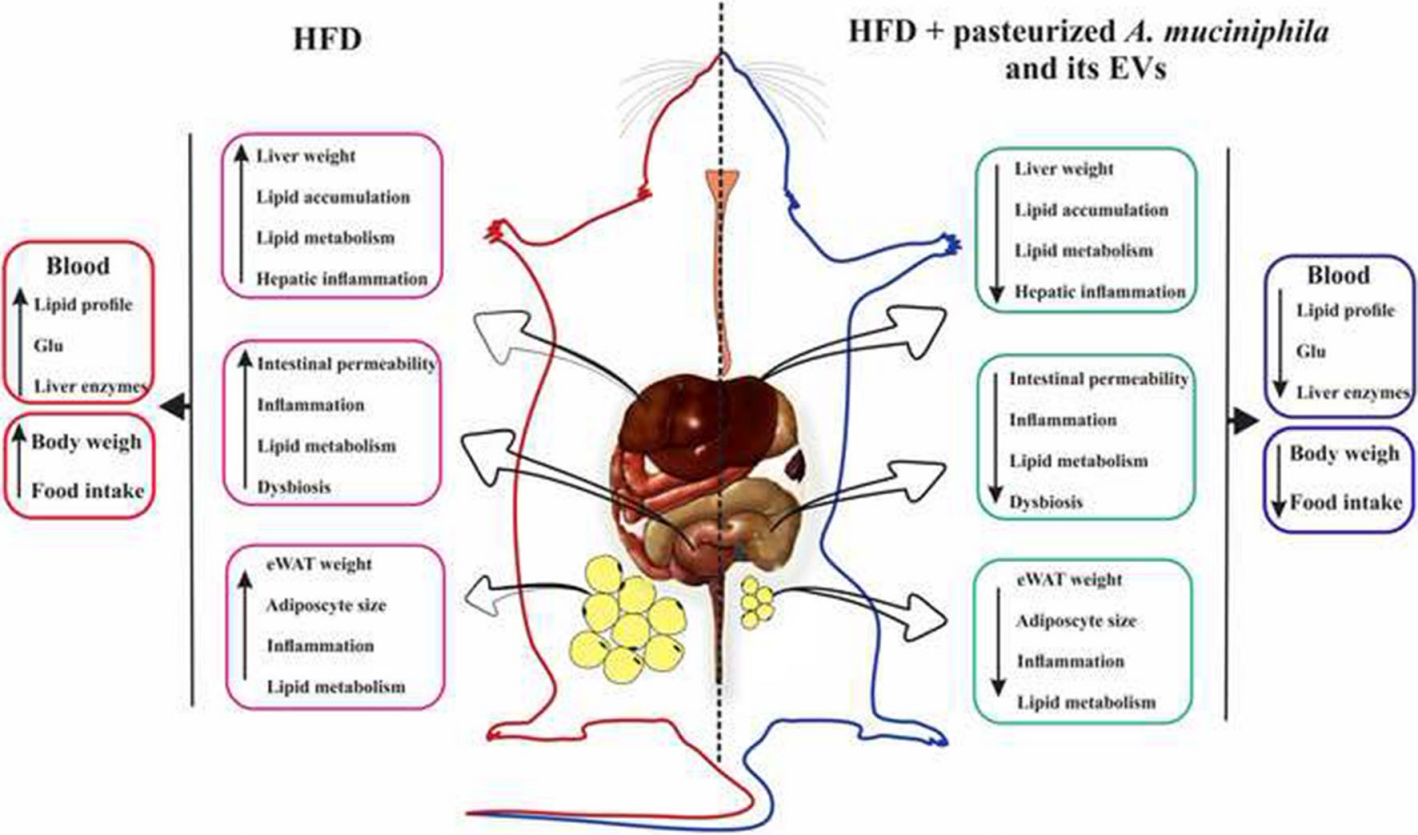

**Supplementary Information:**

The online version contains supplementary material available at 10.1186/s12934-021-01709-w.

## Background

The inter-organ communication is vital for the control and maintenance of energy, metabolism, and immune homeostasis. The phenotypic consequence of disruptions in this type of communication is obesity, which is one of the main public health and medical concerns worldwide [1, 2]. So far, several therapeutic and preventive medications have been used for obesity management, which can increase energy expenditure and reduce fat absorption, energy intake, and appetite [3]; however, these medications have several side effects such as adverse cardiovascular, psychiatric, and gastrointestinal events [4].

Recently, probiotics have received considerable attention as beneficial agents for preventing obesity by manipulating the intestinal microbiota through improved the gut microbiota composition, the colonization resistance phenomenon, and production of various metabolites (e.g., short-chain fatty acids (SCFAs) and vitamins) [5–7]. The intestinal microbiota plays a major role in health and disease by modulating the immune response and host metabolism. Multiple studies have revealed that a high-fat diet (HFD) is correlated with the gut microbiota disturbances, intestinal barrier permeability, onset of intestinal, adipose, and hepatic inflammations, changes in lipid metabolism, and consequently, obesity-induced metabolic disorders [8, 9]. HFD reduces the expression of tight junction proteins and increases intestinal permeability, leading to the transfer of bacterial fragments such as lipopolysaccharides (LPS) into the circulation and induction of metabolic endotoxemia. In addition, an increase in plasma LPS levels affects multiple organs and induces metabolic changes and systemic inflammation. These changes are associated with a significant reduction in the beneficial bacterial population and an increase in pathogenic bacteria in the gut [7]. However, the central mechanism and roles of inter-organ communication in the pathogenesis of obesity, have not been completely clarified.

Growing evidence shows that the gut microbiota plays a vital role in the management of the host metabolism through interplay between metabolic tissues [10]. Therefore, treatment of the gut microbiota by probiotics, postbiotics, and paraprobiotics is considered a potential strategy for the treatment and prevention of obesity [11, 12]. It should be noted that paraprobiotics (cellular structural components) and postbiotics (metabolic products secreted by probiotics) are two forms of non-viable probiotics, when administrated in sufficient amounts, could have beneficial effects on host health [13]. Several strains of probiotics can reverse the HFD-induced adverse effects, including *Akkermansia muciniphila* (*A. muciniphila*), which is widely regarded as a next-generation probiotic [14–16]. Our previous research as well as many other studies have reported the beneficial effects of *A. muciniphila* on energy metabolism, fatty acid (FA) oxidation, inflammation, and gut integrity [15–20]. Considering the sensitivity of *A. muciniphila* to oxygen and its reduced efficacy during administration, recent studies have suggested the use of its inactive form or its derivatives rather than its live form [15, 16, 21, 22].

A recent study revealed that pasteurized *A. muciniphila* had more significant effects on metabolism as compared to its live form and could reduce the weight gain and increase the total energy expenditure [17]. The interaction of the intestinal microbiota with the host and regulation of multiple signaling pathways are triggered by the secretion of nano-sized extracellular vesicles (EVs), which can pass the mucus layers and transfer to peripheral tissues through circulation [22–24]. In this regard, many studies have reported the positive effects of the gut microbiota-derived EVs on the amelioration of several diseases [15, 21, 22]. However, further studies are needed to determine the precise mechanism of the microbiota EVs in the prevention of diseases.

Although several studies have been conducted on the effects of *A. muciniphila* cells, fragments and proteins in various diseases models, no systematic comparison of *A. muciniphila*-derived EVs, pasteurized and live cells have been reported. Therefore, in the present study, we aimed to use the well-defined model of diet-induced obesity to show the best effect of live and pasteurized *A. muciniphila* and its EVs to prevent weight gain, inflammation and lipid metabolism. We also aimed to compare the effects of live and pasteurized *A. muciniphila* and its EVs on the prevention of obesity.

## Results

### Characterization of EVs

The morphology and size of EVs derived from *A. muciniphila* were evaluated by SEM; the EVs were spherical shape and a range of 40–150 nm in size (Additional file 1: Fig. S1).

### *A. muciniphila* prevented increased food intake and plasma metabolic parameters in HFD-fed mice

**Fig. 1:**
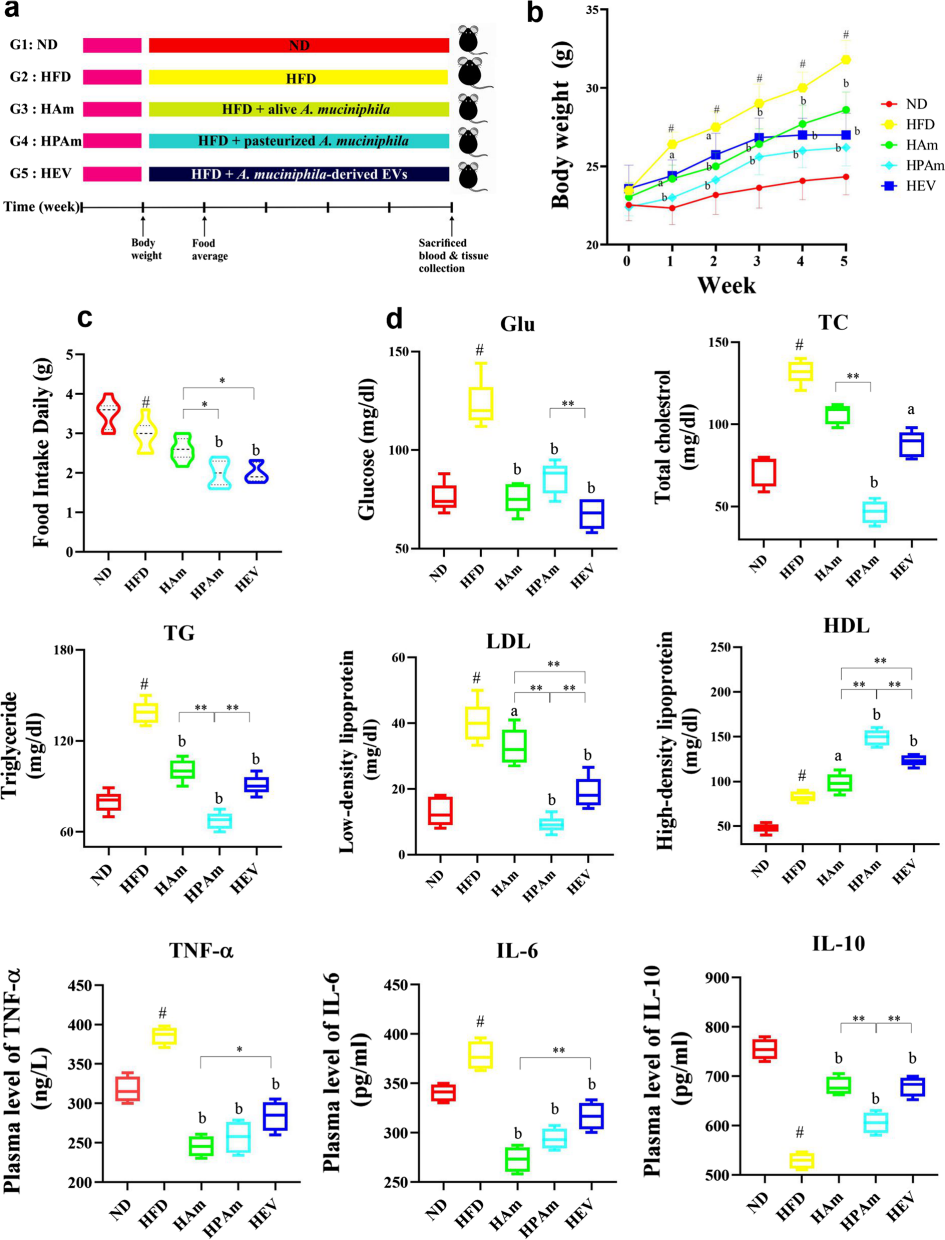
The effects of alive, pasteurized *A. muciniphila* and its EVs on prevention of HFD-induced increase in body weight, food intake and plasma metabolic parameters in HFD-fed mice. **a** Experimental scheme, **b** Body weight, and **c** Average daily food intake in ND, HFD, and treatment groups. **d** The concentration levels of metabolic parameters e.g., Glucose, Triglyceride, Total cholesterol, LDL, HDL, and cytokines (i.e., TNF-α, IL-6, and IL-10) in the plasma of mice. Data are presented as the mean ± SD, N = 7 per group. ^#^*p* < 0.05; HFD vs. ND, ^a^*p* < 0.05; ^b^*p* < 0.01; treatment groups vs. HFD, and *p < 0.05; **p < 0.01; among treatment groups were considered statistically significant, respectively. ND: normal diet + PBS, HFD: high fat diet + PBS, HAm: high fat diet + *A. muciniphila* (10^9^ CFU), HPAm: high fat diet + pasteurized *A. muciniphila* (10^9^ CFU), and HEV: high fat diet + EVs (10 µg protein)

To assess the impact of live and pasteurized *A. muciniphila* and its EVs on preventing obesity, mice were fed an HFD for five weeks without or along with treatment. The body weight of the HFD group was significantly enhanced than the ND group. All treatments prevented HFD-induced body weight gain, while pasteurized form significantly had a better effect than live form (Fig. 1b). Food intake also significantly increased in HFD mice than normal mice, while pasteurized *A. muciniphila* and its EVs significantly decreased food intake (Fig. 1c).

The glucose concentration in all treatments were significantly reduced relative to the HFD group, among which the HEV and HAm groups showed lower concentration. In comparison with ND mice, HFD increased plasma lipids profile level, however, all treatments showed preventive effects on HFD-induced hyperlipidemia. A significant reduction in TC level was observed in the HPAm group. All treatments significantly decreased TG and LDL levels as well as markedly increased the HDL concentration (Fig. 1d).

In the HPAm group, the greatest preventive effects on the plasma metabolic indicators of obesity were observed. The HFD markedly increased the plasma level of pro-inflammatory cytokines (i.e., TNF-α and IL-6) and decreased the level of IL-10 as an anti-inflammatory cytokine, compared to the ND-fed mice. However, all treatments could prevent the HFD-induced inflammation. Notably, live and pasteurized *A. muciniphila* induced the greatest effects on the reduction of pro-inflammatory cytokines, while the highest concentration of IL-10 was found in the HAm and HEV groups. Overall, the EVs and pasteurized *A. muciniphila* exerted more potential preventive effects on obesity, characterized by a decrease in body weight, blood biochemical parameters, and food intake.

### Expression of key genes involved in lipid metabolism and inflammation

**Fig. 2:**
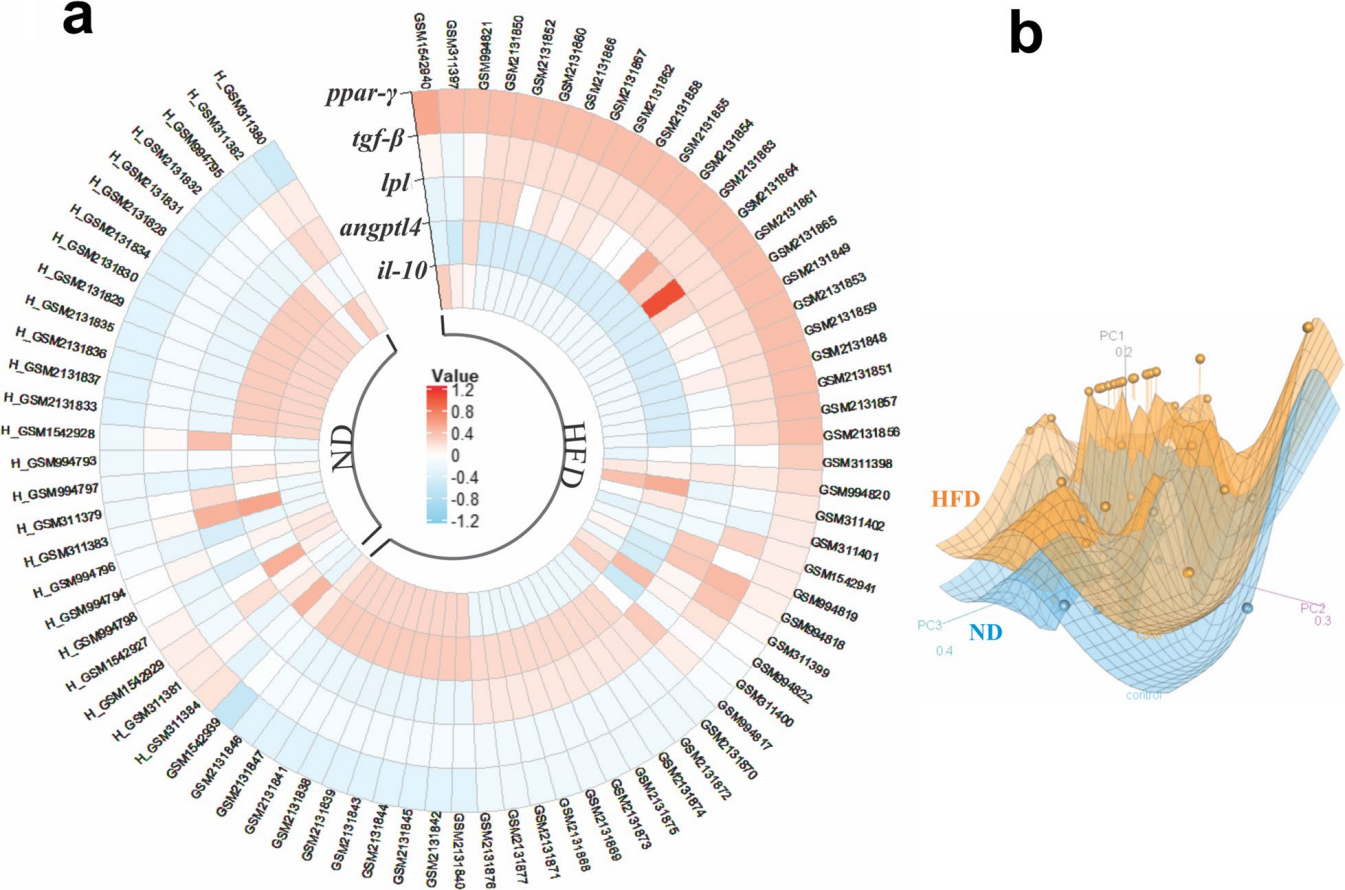
Heatmap and PCA correlation display different hepatic genes involved in fatty liver between HFD and ND mice. **a** Heatmap plot of the expression of genes related to HFD-induced fatty liver in HFD- than ND-fed mice. **b** PCA plot exhibited that the ND group was clustered relatively from the HFD group. HFD and ND group indicated by orange and blue colors respectively

According to our meta-analysis, the expression of these genes in the liver was closely associated with the HFD-induced fatty liver, and they were differentially expressed (P < 0.05). The heat-map plot revealed that lipid metabolism-associated genes (*ppar-*γ and *lpl*) and *tgf-β* were significantly enriched in the HFD group as compared to normal mice, while *angptl4* and *il-10* were lower in the HFD group, compared to normal mice (Fig. 2a). The principal component analysis (PCA) was also applied for the selected genes from all 22 ND and 52 HFD samples in four datasets. The results revealed that the ND group was relatively clustered from the HFD group (Fig. 2b). Since these genes, which play a crucial role in lipid metabolism and inflammation, showed similar significant trends in all datasets, they were selected to examine the effects of live and pasteurized *A. muciniphila* and its EVs on genes associated with HFD-induced fatty liver.

### *A. muciniphila* prevented the onset of fatty liver in HFD-fed mice by modulating lipid metabolism and inflammation

**Fig. 3:**
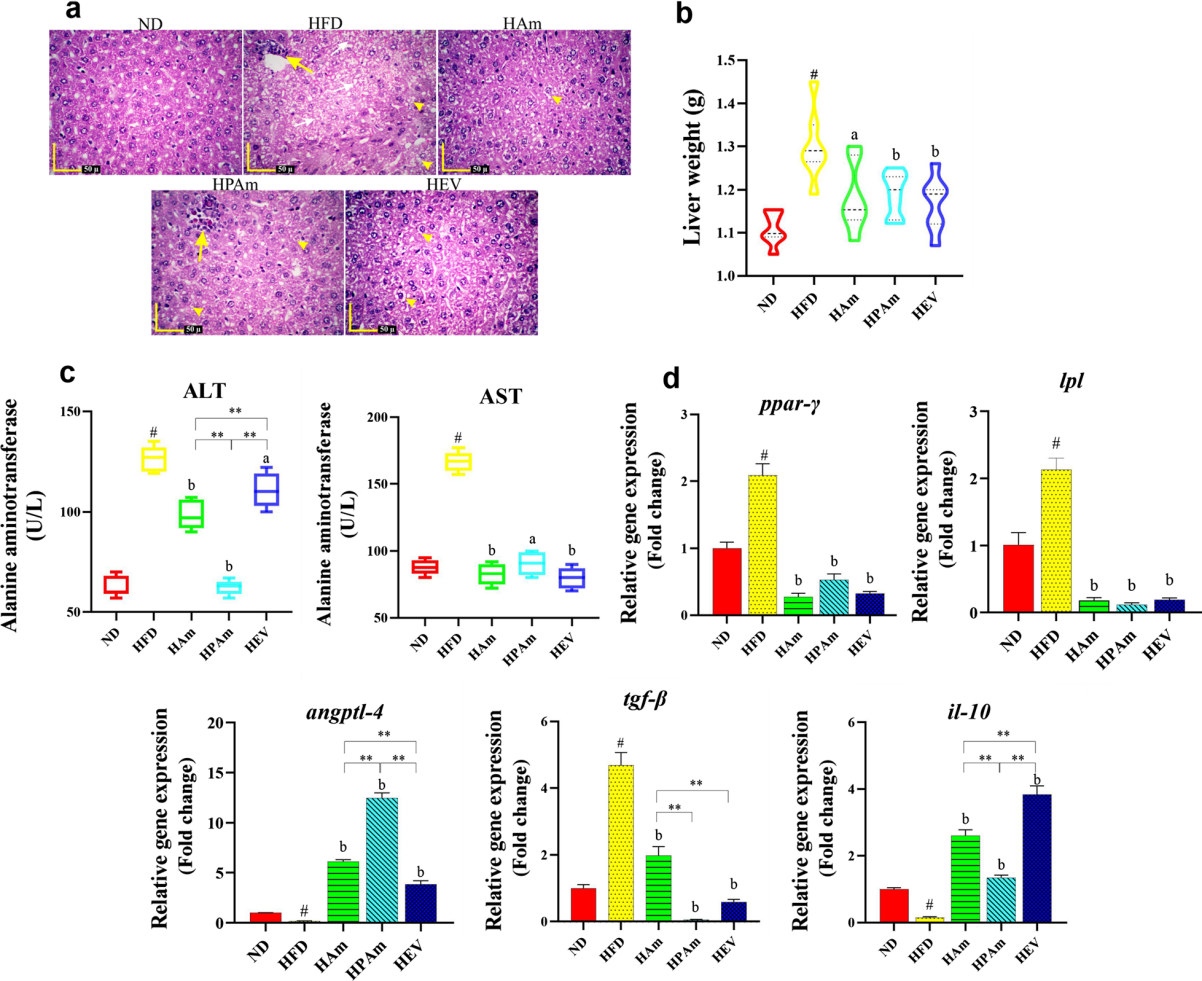
The effects of alive, pasteurized *A. muciniphila* and its EVs on weight, pathology, and expression of liver injury-related genes in the liver of HFD-fed mice. **a** H&E staining of liver section (Yellow arrows: infiltration of inflammatory cells, yellow arrowheads: lipid droplets in microvesicular form, and white arrows: lipid droplets in macrovesicular form, scale bar is 50 µm). **b** Liver weight in ND, HFD, and treatment groups. **c** The concentration of ALT and AST in plasma of mice. **d** Relative mRNA expression of lipid metabolism and inflammation-related genes (*ppar-*γ*, lpl*, *angptl4*, *tgf-β*, and *il-10*). Data are presented as the mean ± SD, N = 7 per group. ^#^*p* < 0.05; HFD vs. ND, ^a^*p* < 0.05; ^b^*p* < 0.01; treatment groups vs. HFD, and *p < 0.05; **p < 0.01; among treatment groups were considered statistically significant, respectively. ND: normal diet + PBS, HFD: high fat diet + PBS, HAm: high fat diet + *A. muciniphila* (10^9^ CFU), HPAm: high fat diet + pasteurized *A. muciniphila* (10^9^ CFU), and HEV: high fat diet + EVs (10 µg protein)

To examine the effects of live or pasteurized *A. muciniphila* and its EVs on fatty liver, we assessed the liver histopathology, liver enzymes, and hepatic lipid and inflammation-related genes. In the histological analysis, a higher infiltration of inflammatory cells was observed in the HFD group than the ND group. Although all treatments prevented the HFD-induced inflammation, a few inflammatory cells were found in the HPAm group. Moreover, excessive accumulation of lipid droplets in both macrovesicular and microvesicular forms was found in HFD-exposed hepatocyte cells, while all treatments prevented and reduced the HFD-induced accumulation of lipid droplets; nevertheless, the HPAm group showed few lipid droplets (Fig. 3a) Overall, live *A. muciniphila* and its EVs exerted better preventive effects on HFD-induced hepatic steatosis and induced a normal morphology similar to the ND group.

HFD enhanced liver weight, however HEV, HPAm, and HAm significantly prevented HFD-induced weight gain. Notably, hepatic weight of HEV and HPAm were lowered, but no significant difference was observed in hepatic weight as compared to HAm group (Fig. 3b). ALT and AST concentrations in the HFD group were highest, while all treatments prevented these alterations, notably, pasteurized *A. muciniphila* had the most effective reduction in ALT level (Fig. 3c).

Following HFD feeding, the expression of hepatic lipid metabolism-related genes, e.g., *ppar-*γ and *lpl,* were upregulated as well as *angptl4* expression was downregulated. Interestingly, all treatments had lipid-associated genes lowering effects. The highest expression level of *tgf-β* was observed in the HFD group, while all treatments prevented this upregulation; however, the remarkable effect on down-regulation of *tgf-β* was observed in the HPAm and HEV as compared to HAm group. In HFD-fed mice, the mRNA level of *il-10* was weakly expressed, however treatments potentially exert anti-inflammatory effect via expression of high *il-10* level (Fig. 3d). It is intriguing to note that the EVs showed the highest anti-inflammatory effect. Altogether, these results showed that *A. muciniphila* could prevent HFD-induced fatty liver.

### *A. muciniphila* administration prevented HFD-induced intestinal barrier disturbance and inflammation

**Fig. 4:**
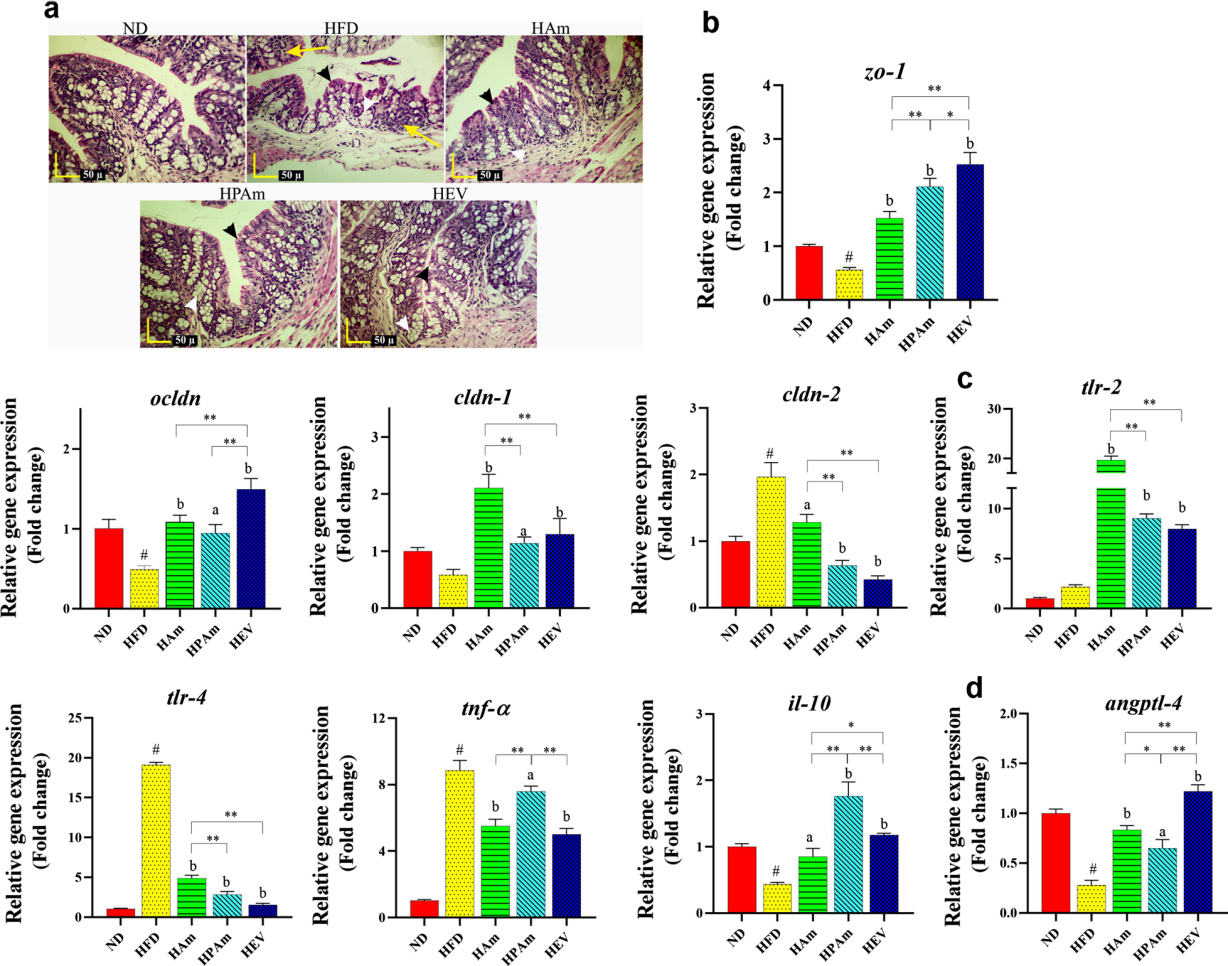
The effects of alive, pasteurized *A. muciniphila* and its EVs on morphology, integrity, and inflammation in the colon of HFD-fed mice. **a** H&E staining of colon section of mice (Yellow Arrows: focal infiltration of inflammatory cells, white arrows: crypt depth, and black arrowheads: mucous thickness, scale bar is 50 µm). **b** The expression of tight junction proteins (e.g., *zo-1, ocldn, cldn-1,* and *cldn-2*), **c** inflammatory-related genes (e.g., *tlr-2, il-10*, *tlr-4,* and *tnf-α*)*,* and **d** *angptl-4* in the colon of mice. Data are presented as the mean ± SD, N = 7 per group. ^#^*p* < 0.05; HFD vs. ND, ^a^*p* < 0.05; ^b^*p* < 0.01; treatment groups vs. HFD, and *p < 0.05; **p < 0.01; among treatment groups were considered statistically significant, respectively. ND: normal diet + PBS, HFD: high fat diet + PBS, HAm: high fat diet + *A. muciniphila* (10^9^ CFU), HPAm: high fat diet + pasteurized *A. muciniphila* (10^9^ CFU), and HEV: high fat diet + EVs (10 µg protein)

Because HFD induced an alteration in morphology, integrity, and inflammation of HFD-fed mice colon, we evaluated colon tissue by H&E staining and qPCR. HFD induced focal infiltration of inflammatory cells in the lamina propria and epithelium in the colon; conversely, no inflammatory reactions were present in all treatments. Moreover, the crypt depth and mucous layer thickness showed a considerable decrease in the HFD group, in contrast, all treatments prevented these changes (Fig. 4a).

HFD reduced intestinal integrity by downregulating. *zo1*, *ocldn*, and *cldn-1* as well as an increase in* cldn-2* expression, whereas modulating these genes were observed after all treatments (Fig. 4b). The highest increase in *zo-1* and *ocldn* was observed in the HEV group, however the greatest upregulation of *cldn-1* was seen in the HAm group. Furthermore, more downregulation in *cldn-2* was observed in the HEV and HPAm groups. These results showed that the modulation of tight junction genes were responsible for suppressing HFD-induced intestinal permeability induced by all treatments.

HFD upregulated the expression of colonic inflammatory markers, whereas all treatments protected against these changes. These observations were accompanied by upregulating *tlr-2* and *il-10* as well as downregulating *tlr-4* and *tnf-α* (Fig. 4c). The EVs had the highest effects on reducing mRNA level of inflammatory genes, while live and pasteurized *A. muciniphila* had more increasing *tlr-2* and *il-10* expression, respectively. These findings indicated that pasteurized *A. muciniphila* and its EVs had a markedly more influence on the protection against HFD-induced intestinal alterations.

To explore the impact of live and pasteurized *A. muciniphila* and its EVs on lipid metabolism, we assessed colonic gene expression of Angiopoietin-like 4 (Angptl4). The colonic expression of *angptl4* reduced in the HFD group, whereas significantly upregulated in treatment groups, among which the HEV group had a better effect (Fig. 4d). Together, the EVs had notable impacts on the prevention of obesity by the modulation of genes involved in adiposity and energy metabolism.

### *A. muciniphila* prevented an increase in adipocyte size, lipid metabolism, and inflammation in adipose tissue of HFD-fed mice

**Fig. 5:**
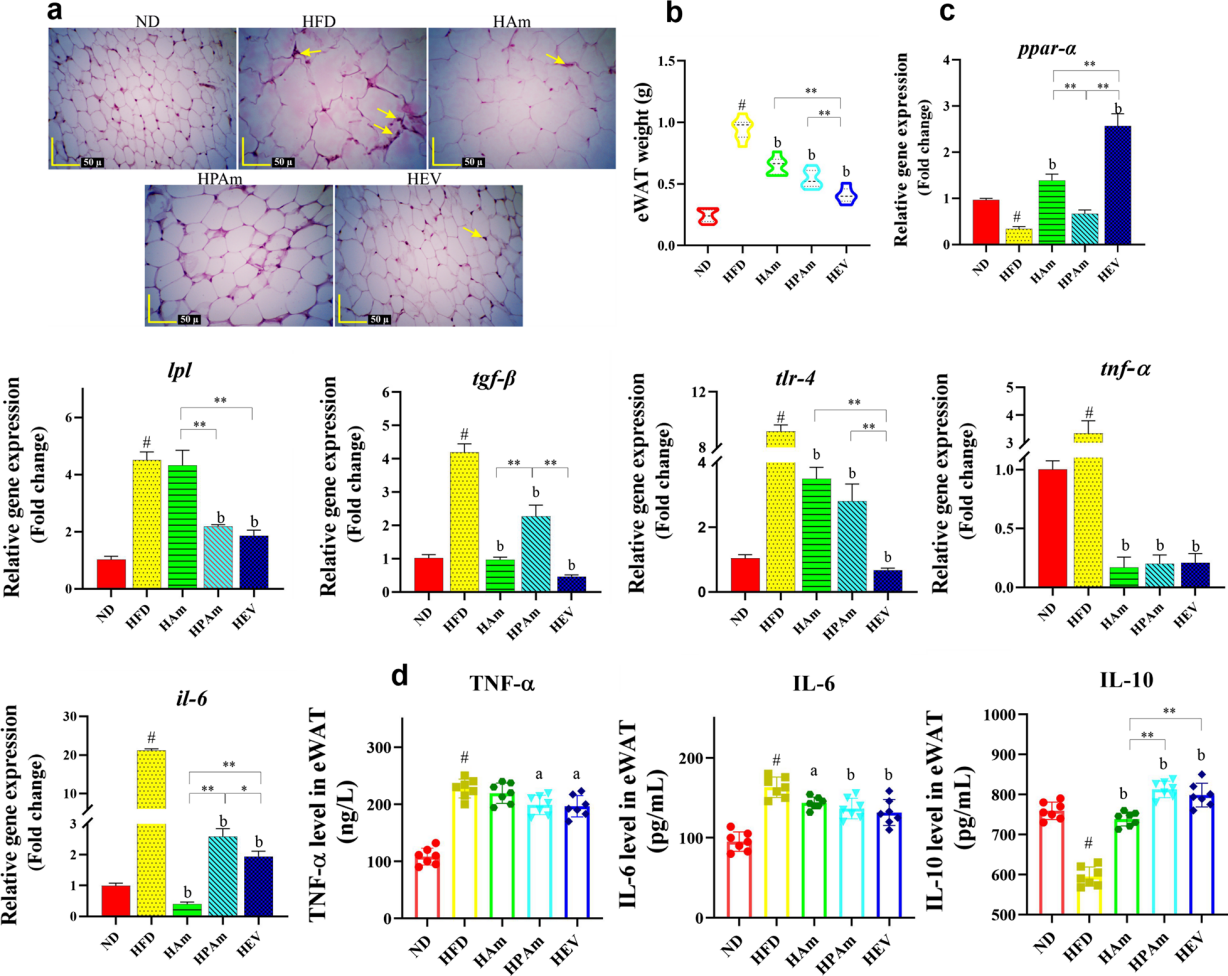
The effects of alive, pasteurized *A. muciniphila* and its EVs on weight, morphology, obesity-related genes, and inflammatory mediators in epididymal white adipose tissue of HFD-fed mice. **a** H&E staining of epididymal white adipose tissue (eWAT) section of mice (Yellow Arrows: infiltration of inflammatory cells, scale bar is 50 µm). **b** eWAT weight in ND, HFD, and treatment groups. **c** The expression of lipid metabolism and inflammation-related genes (e.g., *ppar-α*, *lpl*, *tgf-β*, *tlr-4*, *il-6*, and *tnf-α*) in the eWAT of mice. **d** The concentration of cytokines (i.e., TNF-α, IL-6, and IL-10) in the eWAT of mice. Data are presented as the mean ± SD, N = 7 per group. ^#^*p* < 0.05; HFD vs. ND, ^a^*p* < 0.05; ^b^*p* < 0.01; treatment groups vs. HFD, and *p < 0.05; **p < 0.01; among treatment groups were considered statistically significant, respectively. ND: normal diet + PBS, HFD: high fat diet + PBS, HAm: high fat diet + *A. muciniphila* (10^9^ CFU), HPAm: high fat diet + pasteurized *A. muciniphila* (10^9^ CFU), and HEV: high fat diet + EVs (10 µg protein)

To evaluate whether the live and pasteurized *A. muciniphila* and its EVs had preventive effects on HFD-caused adipose dysfunctions, we assessed eWAT by histopathology analysis, qPCR, and ELISA. The HFD increased the adipocyte size, whereas all treatments exerted preventive effects on this parameter. In the HEV, HPAm, and HAm groups, the adipocyte size decreased; the HEV group exhibited a normal morphology similar to the ND group. Also, HFD increased the infiltration of inflammatory cells in the interstitial tissue than other treatments; however, inflammation was less alleviated in the HAm and HEV groups as compared to the HPAm group (Fig. 5a).

Results of the adipocyte surface area revealed that the HFD group included the maximum average (5613.71 ± 114.29 µm^2^), compared to that in other groups (P < 0.001). Furthermore, adipocyte surface area showed respectively a significant decrease in HEV (1170.19 ± 72.38 µm^2^), HPAm (2349.31 ± 33.45 µm^2^) and HAm (2452.21 ± 124.36 µm^2^) groups, compared to that in HFD group (P < 0.001) (Additional file 1: Fig. S2).

The HFD group showed a higher eWAT weight than ND-fed mice. However, all treatments significantly prevented this weight gain, the EVs had the greatest effect on it (Fig. 5b). These finding exerted that *A. muciniphila* and its EVs play a key role in preventing HFD-induced obesity by affecting eWAT weight.

In comparison with normal mice, the expression of lipid oxidation gene i.e. *ppar-α* was decreased in the eWAT of HFD-fed mice. All treatments could upregulate this expression; however, the HPAm showed no significant change. The EVs had the highest effect on it. Moreover, *lpl* and *tgf-β* were significantly expressed in the HFD group than the ND group. The HPAm and HEV groups had the same effect on the reduction in *lpl* expression, but HAm didn’t significantly effect on it. The significant downregulation of *tgf-β* was observed in all treatment groups, while the EVs and live *A. muciniphila* showed a more noticeable effect.

The overexpression of adipo-inflammatory genes including *tlr-4*, *il-6*, and *tnf-a* were observed in HFD-fed mice, while treatments alleviated adipo-inflammation. The same reduction of *tlr-4* and *tnf-α* expression were observed in all treatments, also the highest reduction in adipose *il-6* mRNA level was observed in the HAm group (Fig. 5c).

Besides, increased TNF-α and IL-6 level and decreased IL-10 level was also observed in the HFD group, while all treatments prevented HFD-induced adipo-inflammation. Of note, pasteurized *A. muciniphila* and its EVs induced the highest effects on the concentrations of inflammatory mediators (Fig. 5d). These results suggested that the postbiotic and paraprobiotic *A. muciniphila* could prevent HFD-induced adipo-inflammation.

### *A. muciniphila* prevented HFD-induced gut dysbiosis by balancing the microbial population

**Fig. 6:**
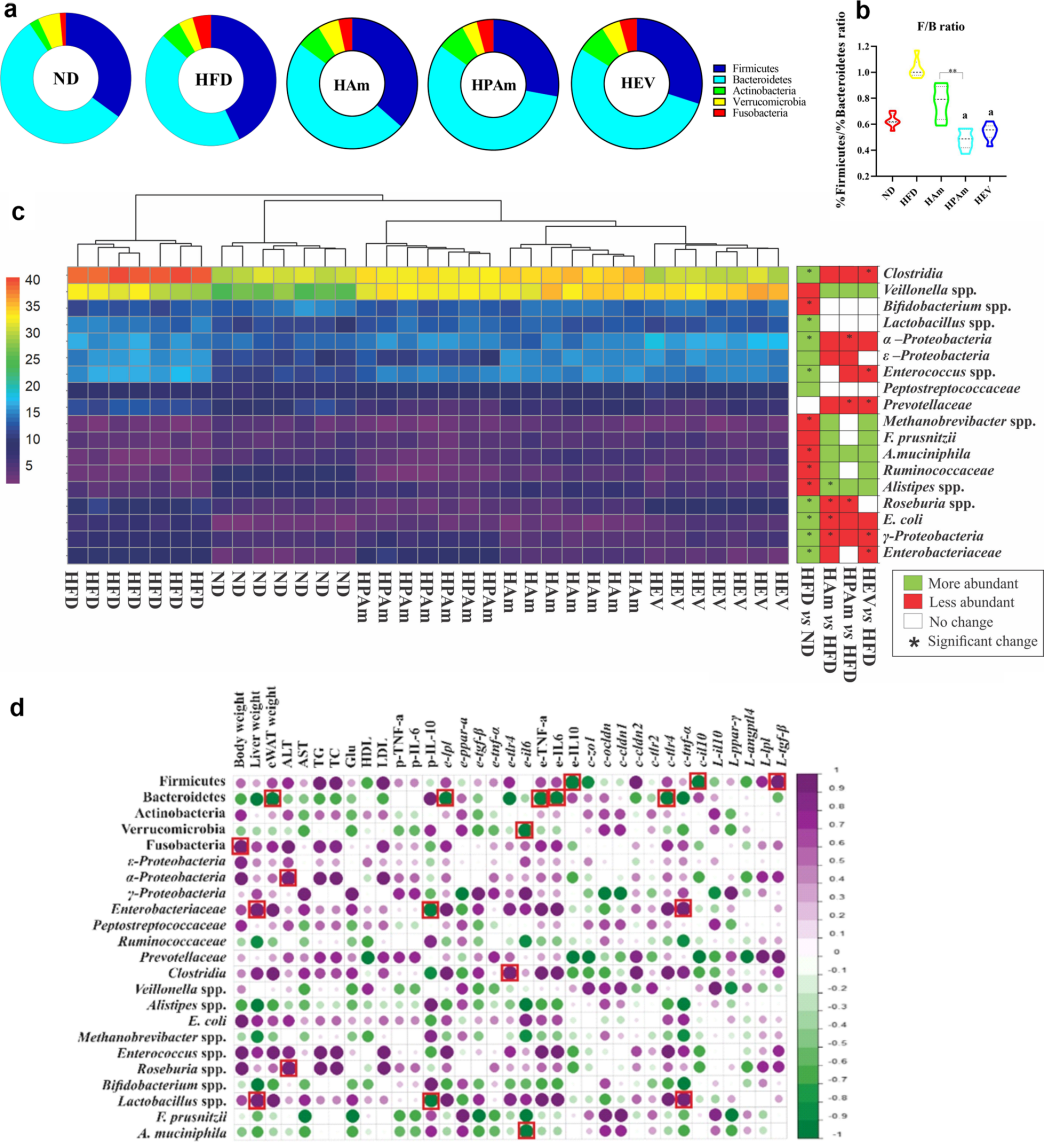
The effects of live, pasteurized *A. muciniphila* and its EVs on in gut microbiota pattern. The relative percentage of **a** phylum abundance and **b** Firmicutes to Bacteroidetes ratio. **c** Heat map of gut microbiota abundance at Family/Class/Genus level in per mouse. **d** The correlation between gut microbiota and obesity-related indexes (Red square: significant correlation). N = 7 per group. ^#^*p* < 0.05; HFD vs. ND, ^a^*p* < 0.05; ^b^*p* < 0.01; treatment groups vs. HFD, and *p < 0.05; **p < 0.01; among treatment groups were considered statistically significant, respectively. ND: normal diet + PBS, HFD: high fat diet + PBS, HAm: high fat diet + *A. muciniphila* (10^9^ CFU), HPAm: high fat diet + pasteurized *A. muciniphila* (10^9^ CFU), and HEV: high fat diet + EVs (10 µg protein)

To explore whether *A. muciniphila* can modulate HFD-mediated gut microbiota dysbiosis, we quantified the microbial composition in gut of mice through 16S rRNA gene-targeted phylum- and group-specific primers (Additional file 1: Table S3) by real-time PCR. HFD significantly augmented Fusobacteria and Firmicutes as well as reduced Bacteroidetes, Actinobacteria, and Verrucomicrobia than the ND group. All treatments affected the gut microbiota composition. The Firmicutes abundance decreased in the HPAm and HEV groups, while the Verrucomicrobia abundance increased in the HAm group (Fig. 6a). On the other hand, pasteurized *A. muciniphila* and its EVs significantly decreased the Firmicutes/Bacteroidetes ratio (Fig. 6b). The heatmap represents a comparison of the relative abundance of bacteria at class, family, and genus levels among the experimental groups (Fig. 6c). There was a significant increase in γ-/ε-/α-Proteobacteria, Enterobacteriaceae, and Clostridia abundance as well as decreased Ruminococcaceae in the HFD group than the ND group. After five weeks, there was a significant decline in α-Proteobacteria in the HPAm group, γ-Proteobacteria in the HAm and HEV groups, Prevotellaceae in the HPAm and HEV groups, and Clostridia in the HEV group. At genus level, the gut microbiota of the HFD group contained the highest level of *Roseburia* spp., *Enterococcus* spp., *Lactobacillus* spp., and *E. coli* and the lowest level of *Veillonella* spp., *Alistipes* spp.*, Bifidobacterium* spp., *Methanobrevibacter* spp.*,* and *A. muciniphila*. Interestingly, live *A. muciniphila* was the most effective in preventing these HFD-induced dysbiosis, which was accompanied by increasing *Alistipes* spp. and *A. muciniphila* and decreasing *Roseburia* spp. and *E. coli*. Pasteurized *A. muciniphila* also significantly reduced the abundance of *Roseburia* spp. Overall, *A. muciniphila* could avoid HFD-induced dysbiosis by decreasing obesity-related pathobiont bacteria and increasing health-related gut microbiota.

### Effects of *A. muciniphila* on correlation between the gut microbiota abundance and obesity-related indices

The correlations between gut microbiota and obesity-related indices demonstrated that Firmicutes was negatively correlated with adipose IL-10 level and colonic *il-10* expression, whereas it had positive association with hepatic *tgf-β* expression. Bacteroidetes level was negatively correlated with the eWAT weight, the adipose level of TNF-α and IL6, the expression of adipose *lpl*, and colonic *tlr-4*. There was reverse correlation between body weight and Fusobacteria abundance, which had the highest relative abundance in the HFD group. Verrucomicrobia abundance was a negative correlation with adipose *il-6* mRNA level*.* Besides the liver weight, the colonic *tnf-α* mRNA level had a significant positive association with the abundance of Enterobacteriaceae and *Lactobacillus* spp., while the abundance of these bacteria had a negative association with the plasma IL-10 level. Moreover, the Clostridia abundance was positively associated with the adipose *tlr-4* mRNA level, while *A. muciniphila* had a negative correlation with the *il-6* expression in the eWAT*.* ALT was positively correlated with pathobiont bacteria, such as *Roseburia* spp. and α-Proteobacteria (Fig. 6d). These findings revealed that the administration of *A. muciniphila* could play a beneficial role in the modulation of host responses by affecting the HFD-induced intestinal dysbiosis.

## Discussion

Numerous studies have indicated that several probiotics can prevent HFD-induced obesity [5, 11, 12, 15], while the exact mechanism of probiotics remains unclear. In this study, we carried out a comparative analysis of the effects of live and pasteurized *A. muciniphila* and its EVs on the HFD-fed mice to understand the possible mechanisms underlying their preventive effects on obesity.

Functional studies have reported that HFD increases the body weight, metabolic tissue weight, adipocyte size, and hepatic lipid droplets. On the other hand, probiotics can inhibit the weight gain and other HFD-induced effects [16, 25]*.* We found that all treatments reduced the body, eWAT, and liver weight gain, in addition to the adipocyte size and hepatic lipid droplets in HFD-fed mice; however, the efficacy of treatments varied. Pasteurized *A. muciniphila* and its EVs showed greater effectiveness than other treatments. Similarly, the effects of *A. muciniphila* and its EVs in HFD-fed mice revealed that this bacterium and its EVs could ameliorate obesity by reducing the body weight gain, fat accumulation, and pathological abnormalities [14, 15, 22]. Besides, an earlier study demonstrated that pasteurized *A. muciniphila* had greater effects on the fat mass loss and obesity amelioration compared to its live form [16]. Since the gut microbiota is critical for the management of body weight, our results showed that probiotics, paraprobiotics, and postbiotics may have potential effects on preventing diet-induced obesity.

Besides, probiotics can affect the plasma lipid profile and inflammatory cytokines and ameliorate obesity in HFD-fed animals [15, 26]. We found that all treatments improved the metabolic parameters and prevented the onset of obesity; notably, pasteurized *A. muciniphila* showed greater effects on these parameters. In agreement with our study, previous studies revealed that administration of live and pasteurized *A. muciniphila* could reduce the level of cholesterol in animal and human models [16, 27]. Moreover, we reported the reducing effects of *A. muciniphila* and its EVs on the metabolic parameters in obese mice [15]. These findings indicated that both forms of *A. muciniphila* and its EVs play a preventive role in the onset of HFD-induced obesity and its related complications.

Substantial evidence corroborates that obesity is associated with intestinal dysfunction and low-grade inflammation. Therefore, regulation of obesity-related inflammation and restoration of the impaired intestinal barrier function have been proposed as potential therapeutic targets in the past few decades [28, 29]. Our findings indicated the greatest increase in the intestinal permeability and inflammatory mediators, besides the lowest mucus thickness and crypt depth in the HFD group, while all treatments prevented these changes. The mechanism of HFD includes reducing intestinal integrity, penetrating LPS into lamina propria, stimulating the immune system, and inducing low-grade inflammation [30]. Thus, all treatments prevented HFD-induced morphological changes by decreasing gut permeability and ameliorating LPS-induced inflammation.

Remarkably, the pasteurized *A. muciniphila* and its EVs completely suppressed the HFD-induced intestinal inflammation in HFD-fed mice and protected the intestinal permeability. In this regard, previous studies on pasteurized and live *A. muciniphila* showed that HFD-induced intestinal permeability was alleviated following both treatments by affecting the expression of tight junction components [16] and colonic morphological features [14, 31]. Moreover, research on *A. muciniphila* EVs demonstrated their beneficial effects on the obesity-induced gut permeability [15, 22], DSS-induced colitis [21], and Caco-2 cell line [32]. Overall, these findings suggest that probiotics can strengthen the intestinal barrier integrity, reduce inflammation, and consequently, prevent metabolic disorders, which might be due to a close link between probiotics and intestinal cells.

In addition, we observed that all treatments significantly upregulated the colonic expression of Angptl-4, while HFD suppressed it. Similarly, it has been shown that *A. muciniphila* and its EVs, in addition to *Lactobacillus rhamnosus* CNCMI-4317, upregulated *Angptl-4* in obese mice and the colon cell line, respectively [15, 33]. Therefore, probiotics and their vesicles may have positive effects on the intestinal homeostasis and may prevent obesity.

According to previous studies, the HFD-induce leaky gut is strongly linked to key metabolic dysfunctions in the white adipose tissue by triggering inflammation and lipid metabolism; also, lipid is redistributed in ectopic organs [14, 34]. Our results indicated that HFD increased the eWAT weight, inflammation, and energy homeostasis disturbances; conversely, all treatments protected these alterations. The EVs showed the greatest effects on the adipocyte size, eWAT weight, and lipid homeostasis, compared to the other treatments. Besides EVs, pasteurized *A. muciniphila* had remarkable effects on the adipose inflammatory mediators. It has been shown that *A. muciniphila* improves the metabolic status in obese mice by reducing the expression [31] and production [35] of adipose inflammatory cytokines. During obesity, adipose tissue remodeling occurs, which causes adipocyte enlargement, extracellular matrix components accumulation, and infiltration of pro-inflammatory macrophages, thereby modulating adipose tissue remodeling could prevent obesity [36]. Our finding demonstrated that all treatments reduced adipo-inflammation and adipocyte size, which may be related to the prevention of HFD-induced adipose tissue remodeling in obese mice.

Recent investigations have confirmed the regulatory effects of *A. muciniphila* on energy homeostasis in obese mice by reducing the energy absorption and increasing the fecal energy excretion [16, 17]. Besides, pasteurized *A. muciniphila* could modulate the fat mass and prevent diet-induced obesity [16]. These findings indicate that active and inactive probiotics and their derivatives can prevent HFD-induced inflammation and maintain the energy balance. This finding supports the potential health benefits of probiotic, paraprobiotic, and postbiotic *A. muciniphila* in the prevention of obesity.

HFD, by increasing the intestinal permeability, leads to the transfer of bacterial LPS and other components to the liver through the portal vein and results in hepatic inflammation [9]. Also, the HFD-induced adipocyte dysfunction increases the free fatty acids export from the adipose tissue to the liver and leads to fatty liver development [37]; however, probiotics can prevent and ameliorate non-alcoholic fatty liver disease (NAFLD) [38]. In this study, we found a significant increase in the plasma concentrations of ALT and AST, liver weight, fat content, and inflammatory status in the liver sections of HFD-induced mice; nevertheless, all treatments alleviated these liver alterations. It seems that live and pasteurized *A. muciniphila* reduce the level of liver injury-related enzymes in obese mice and individuals [27, 39, 40]; the EVs and pasteurized form of *A. muciniphila* also showed more significant effects on the prevention of fatty liver. These findings support the hypothesis that non-viable probiotics can be more effective in counteracting obesity-related fatty liver disease due to a HFD-related leaky gut.

Generally, HFD causes changes in the expression of hepatic genes involved in lipid metabolism and inflammation. Moreover, the association of *lpl* overexpression and accumulation of liver triglycerides with NAFLD has been shown in the literature, while the overexpression of hepatic Angptl4 seems to inhibit LPL in peripheral tissues [41, 42] and play an important role in the protection of fat accumulation [43] and FA-related inflammation [44]. Our results, in line with our meta-analysis, showed that HFD changed the expression of fatty liver-related genes by upregulating *ppar-γ*, *lpl*, and *tgf-β* and downregulating *angptl4* and *IL-10* in the liver of mice; on the other hand, all treatments could modulate their expression.

According to a previous study, probiotic supplementation reduced the hepatic and adipose *lpl* expression in HFD-fed mice and ameliorated obesity [45]. In the present study, we found the beneficial effects of all treatments on preventing fatty liver, as indicated by the improved lipid metabolism, inflammation, and fibrosis genes in the liver. Consistent with our findings, *A. muciniphila* had protective effects against fatty liver by reducing the FA synthesis and inflammation in the liver of obese mice [46]. Moreover, animal and cell line studies demonstrated that live and heat-killed probiotics can reduce the level of hepatic inflammatory and fibrotic genes [47, 48]. These findings revealed that probiotic, postbiotic, and paraprobiotic *A. muciniphila* had protective effects against fatty liver disease, probably through the MAMPs and bioactive components.

Several studies have shown a strong interaction between the gut microbiota disruptions and the onset of obesity [49, 50]. The increased abundance of Firmicutes and the decreased level of Bacteroidetes have been reported in obese mice [51], which suggests a possible link between Firmicutes and increased calorie intake [52]. We found the increased level of Firmicutes and the reduced level of Bacteroidetes in the HFD group, while the Firmicutes abundance decreased in both HPAm and HEV groups. In line with other studies [53, 54], we found that the Firmicutes/Bacteroidetes (F/B) ratio remained unchanged in the HFD and ND groups, while a higher F/B ratio in obese compared to lean individuals were reported in other studies [55, 56]. According to controversial data about this ratio, it is difficult to link the ratio with determining health status and consider it as a hallmark of obesity [57]. In our study, all treatments decreased this ratio, although it was more significant in the HPAm and HEV groups. Moreover, recent investigations have demonstrated that Actinobacteria and Verrucomicrobia have negative and positive correlations with obesity, respectively [58, 59]. We also showed similar results in the HFD group, while a significant increase in the Verrucomicrobia abundance was seen in the HAm group.

In the present study, the abundance of some pathobiont bacteria, such as the γ/ε/α-proteobacteria, Enterobacteriaceae, Prevotellaceae, and Clostridia*,* increased in the HFD group, while the treatments could reduce their abundance. The high abundance of harmful bacteria has been also reported in obese patients [60]. On the contrary, the abundance of Prevotellaceae seems to reduce in compound probiotic-treated rats [61], which is similar to our results in the HPAm and HEV groups. Moreover, the decreased level of Enterobacteriaceae was reported in *Lactobacillus paracasei* HII0-treated obese rats [62]. These observations demonstrated that HFD might cause an imbalance in the gut microbiota, increase the level of harmful bacteria, and consequently increase LPS-induced inflammation; on the other hand, probiotics could modulate these alterations.

Many studies have reported a reduction in the abundance of Ruminococcaceae, *Bifidobacterium* spp., *F. prausnitzii*, and *A. muciniphila* as beneficial bacteria in the HFD group [63, 64]. Similarly, we found the same reduction in the HFD group, although the level of *A. muciniphila* increased in the HAm group. These findings revealed the beneficial effects of probiotics on the restoration of health-promoting microbiota. In this regard, a human study indicated a significant decline in the abundance of *Alistipes* spp. and an increase in *Roseburia* spp. in obese individuals [65]. We found similar results in the HFD group, whereas both forms of *A. muciniphila* normalized the level of *Roseburia* spp. close to the ND group. In this study, the Firmicutes abundance was negatively correlated with the adipose *il-10* level and colonic *il-10* expression, while it was positively associated with the hepatic expression of *tgf-β*. Similar to our results, a positive correlation between Firmicutes and inflammation was observed in a previous study [66], which was due to SCFA hydrolysis by Firmicutes-secreted enzymes [67]. The increased abundance of Fusobacteria and decreased Bacteroidetes were significantly associated with the body weight and eWAT weight, respectively.

Moreover, the level of Bacteroidetes was negatively correlated with colonic inflammation, pro-inflammatory cytokines, and lipid metabolism in the eWAT. Similarly, an inverse correlation have been reported between the level of Bacteroidetes and body fat gain [68] and inflammatory mediators [66]. The negative correlation with inflammation could be due to secreting SCFA (acetate and propionate) [69] or its polysaccharides [70]. Also, the abundance of *Enterobacteriaceae* and *Lactobacillus* spp. was positively correlated with the liver weight and colonic inflammation; conversely, it had a negative correlation with the plasma anti-inflammatory cytokines. The adipose inflammation was positively correlated with the abundance of Clostridia and negatively associated with the abundance of Bacteroidetes, Verrucomicrobia, and *A. muciniphila*.

Based on the present findings, the concentration of ALT was positively associated with the abundance of pathobiont bacteria, such as α-proteobacteria and *Roseburia* species. In this regard, several studies have shown a correlation between the increased level of pathobiont bacteria in the gut and obesity-related indices [49, 71, 72]. These observations reveal that changes in the gut microbiota pattern have destructive effects on metabolic-related biomarkers and body weight, while preventing these alterations by probiotics, postbiotics, and paraprobiotics may be promising for the prevention of obesity.

## Conclusion

The administration of live and pasteurized *A. muciniphila* and its EVs could prevent several complementary mechanisms involved in obesity, such as increased obesity-related indices, elevated inflammatory status in the HFD-fed mice. Notably, pasteurized *A. muciniphila* and its EVs are more likely to be accounted for the preventive effects than its live form. All treatments could also modulate the relative abundance of some genera (by increasing beneficial microbiota and inhibiting the growth of pathobiont bacteria) and maintain a healthy intestinal homeostasis, reduce obesity, and promote health. The present findings suggest that pasteurized *A. muciniphila* as a paraprobiotic agent and its EVs as postbiotic agents can be new preventive strategies against obesity.

## Methods

### Bacterial strains and culture conditions

*A. muciniphila* MucT (ATCC BAA-835) was grown in a basal mucin-based medium as previously described [73]. After the bacterial density reached an OD_600_ of 1, the pellets were removed by centrifugation, washed twice, and re-suspended with anaerobic PBS. The remaining supernatant was used for EVs extraction. Pasteurization of *A. muciniphila* was performed at 70 °C for 30 min.

### Preparation of *A. muciniphila* EVs

Following filtering of the supernatant, EVs were extracted in an ultracentrifuge (Beckman, Germany) at 200 000*g* for 2 h at 4 °C [21]. The final pellets were re-suspended in PBS and stored at − 80 °C. The morphology assessment of EVs and molecular weights of proteins were performed using Scanning Electron Microscopy (SEM) and SDS-PAGE, respectively, as detailed in the Additional file 1: Additional methods.

### Animal and experimental setup

All animal experiments were performed in accordance with the Animal Experiment Committee of Pasteur Institute of Iran guidelines for the care and use of laboratory mice. For the prevention study, 35 eight-week-old male C57BL/6 mice were obtained from the Pasteur Institute of Karaj (Iran). Mice were individually housed with ad libitum access to food and autoclaved water.

(22–24 °C, 40–60% humidity, and 12 h light/dark). After one week of acclimation with normal diet (ND) (A03, safe diet, France), mice were randomly separated into five groups (n = 7), based on best results of our previous study [15]. As shown in Fig. 1a, mice were fed HFD (260 HF, 60% energy from butter (Kcal/kg), safe diet, France) along with treatments for five weeks includes: (1) ND (standard diet + 200 µl PBS) (as control); (2) HFD (HFD + 200 µl PBS) (as control); (3) HAm (HFD + 10^9^ CFU/200 µl live *A. muciniphila*); (4) HPAm (HFD + 10^9^ CFU/200 µl pasteurized *A. muciniphila*); and (5) HEV (HFD + 10 µg protein/200 µl EVs). Body weight was measured weekly and food was monitored daily. Blood, epididymal adipose (eWAT), liver, and colon tissues were collected and stored at − 80 °C. In addition, eWAT, liver, and colon are saved for histological analysis.

### Serological and histological analysis

Fasting blood glucose (Glc), total cholesterol (TC), low-density lipoprotein (LDL), high-density lipoprotein (HDL), triglyceride (TG), alanine aminotransferase (ALT), and aspartate aminotransferase (AST) levels were measured using a commercial kit (Bioclin-Quibasa, Brazil). Moreover, TNF-α, IL-6, and IL-10 cytokines level in plasma and eWAT were quantified by using ZellBio GmbH ELISA kit (Germany) according to the manufacturer’s instructions. For histological evaluation, the tissues were stained with hematoxylin and eosin (H&E). Dino-lite digital lens, Dino Capture 2 software (AnMo Electronics Corp., Taiwan), and light microscope were used for histopathological analysis, as detailed in the Additional file 1: Additional methods. All test was performed in duplicate.

### Computation of selected gene expression

The National Centre for Biotechnology Information Gene Expression Omnibus (GEO) was used to download gene expression datasets. Our inclusion criteria for accessing appropriate datasets as follow: Studies in which: (1) mice fed HFD for 4–6 weeks; (2) the number of samples for each group (i.e., ND and HFD) must be more than one; (3) using liver tissue; and (4) assessed the expression of all selected genes. A total of four datasets (Additional file 1: Table S1) were obtained that qualified for our study: The R (version 3.6.1) statistical computing environment and BioConductor (version 3.12) were used for gene microarray analyses. Prior to meta-analysis, each dataset was normalized and subsequently transformed with base 2 log. Based on the annotation table for each respective dataset, the probeset identifiers converted into their corresponding Gene Symbol. For multiple probes matching to the same gene, the mean factor of the probes was considered as the final expression value of the gene. Data merging has conducted to correct batch effects using ComBat function of SVA package [74]. Then, we survey the expression of all selected genes across the four datasets by using limma package (version 3.40.6).

### Analysis of target genes in colon, adipose, and liver by quantitative real-time PCR

Total RNA was isolated by Trizol reagent (Bio Basic, Canada). The gDNA was removed by DNase I (Qiagen) then RNA was reverse transcribed using PrimeScript RT Reagent Kit (Takara). Real-time PCR was performed using SYBR Premix Ex Taq II (Takara). The target gene expression was normalized to the housekeeping gene *rpl-19* in colon and liver and *hprt-1* in adipose tissue. A sequence of primers used in this study is shown in Additional file 1: Table S2.

### Analysis of gut microbiota abundance in stool by quantitative real-time PCR

Fecal DNA were extracted using a QIAamp Fast DNA Stool Mini Kit (Qiagen, USA) according to the manufacturer's instructions. Real-time PCR was perfomed by RealQ Plus Master Mix Green (Amplicon, Denmark). The ΔC_T_ method was used to measure each primer efficiency [30]. Conversion of C_T_ value to bacterial communication percentage was performed by using percentage formula as previously described  [75]. A sequence of primers used in this experiment is shown in Additional file 1: Table S3.

### Statistical analysis

Statistical analysis was performed by GraphPad Prism 8.0 (GraphPad Software Inc, CA, USA), the one-way ANOVA followed by Tukey’s post hoc test and Kruskal–Wallis test was used where needed. Moreover, the two-way ANOVA test with a Tukey post-hoc test was used to evaluate the effect of treatments on body weight. The correlations between the gut microbiota and obesity-related indices were evaluated by Spearman’s correlation test. Results are presented as the mean ± standard error and P-value of less than 0.05 was regarded as statistically significant.

## Supplementary Information

**Additional file 1.** Tables S1, S2, and S3. Fig. S1 and S2. Additional methods.

## Data Availability

All data generated or analyzed during this study are included in this published article and its additional file..

## Abbreviations

HFD: High fat diet
ND: Normal diet
EVs: Extracellular vesicles
ANOVA: Analysis of variance
AST: Aspartate aminotransferase
ALT: Alanine aminotransferase
TG: Triglyceride
TC: Total cholesterol
LDL: Low-density lipoprotein
HDL: High-density lipoprotein
FA: Fatty acid
Glc: Glucose
eWAT: Epididymal white adipose tissue
GEO: Gene expression omnibus
TNF: Tumor necrosis factor
IL: Interleukin
TGF: Transforming growth factor
ZO1: Zonula occludens 1
OCLDN: Occludin
CLDN: Claudin
TLR: Toll-like receptor
PPAR: Peroxisome proliferator-activated receptors
Angptl4: Angiopoietin-like 4
LPL: Lipoprotein lipase
H&E: Hematoxylin and eosin
NAFLD: Non-alcoholic fatty liver disease
