# Supplementary material for: Extracellular vesicles and pasteurized cells derived from Akkermansia muciniphila protect against high-fat induced obesity in mice
Source: Microb Cell Fact. 2021 Dec 4;20:219. doi: 10.1186/s12934-021-01709-w (PMC8645101; doi:10.1186/s12934-021-01709-w)
Supplement: Supplementary file 1 — Additional file 1. Tables S1, S2, and S3. Fig. S1 and S2. Additional methods. [file 12934_2021_1709_MOESM1_ESM.docx]

**Extracellular vesicles and pasteurized cells derived from *Akkermansia muciniphila* protect against high-fat induced obesity in mice**

Fatemeh Ashrafian^1,2^, Shahrbanoo Keshavarz Azizi raftar^1^, Arezou Lari^3^, Arefeh Shahryari^1^, Sara Abdollahiyan^1,4^, Hamid Reza Moradi^5^, Morteza Masoumi^1,6^, Mehdi Davari^1,6^, Shohreh khatami^7^, Mir Davood Omrani^8^, Farzam Vaziri^1,6^, Andrea Masotti^9^, Seyed Davar Siadat^1,6*^

1 Microbiology Research Center (MRC), Pasteur Institute of Iran, Tehran, Iran

2 Clinical Research Department, Pasteur Institute of Iran, Tehran, Iran

3 Systems Biomedicine Unit, Pasteur Institute of Iran, Tehran, Iran

4 Basic and Molecular Epidemiology of Gastrointestinal Disorders Research Center, Research Institute for Gastroenterology and Liver Diseases, Shahid Beheshti University of Medical Sciences, Tehran, Iran.

5 Department of Basic sciences, School of veterinary Medicine, Shiraz University, Shiraz, Iran

6 Department of Mycobacteriology and Pulmonary Research, Pasteur Institute of Iran, Tehran, Iran

7 Department of Biochemistry, Pasteur Institute of Iran, Tehran, Iran

8 Department of Medical Genetics, Faculty of Medicine, Shahid Beheshti University of Medical Sciences, Tehran, Iran

9 Research Laboratories, Children's Hospital Bambino Gesù-IRCCS, Rome, Italy.

*** Correspondence: Seyed Davar Siadat** [**d.siadat@gmail.com**](mailto:d.siadat@gmail.com) **ORCID ID: 0000-0002-7288-8112**

**
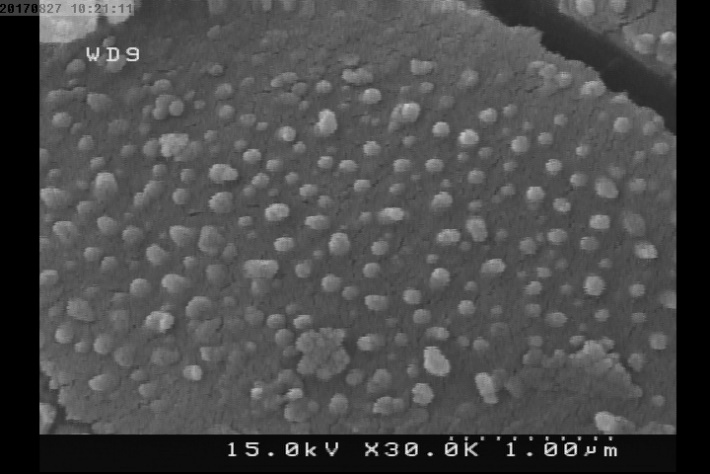
**

**Fig. S1** Morphologic characterization of EVs derived from *A. muciniphila.* Scanning electron micrograph image of *A. muciniphila*-derived EVs. The EVs was in different sizes (a range of 40 to 150 nm) and vesicle-like structures. Scale bar 1 µm.


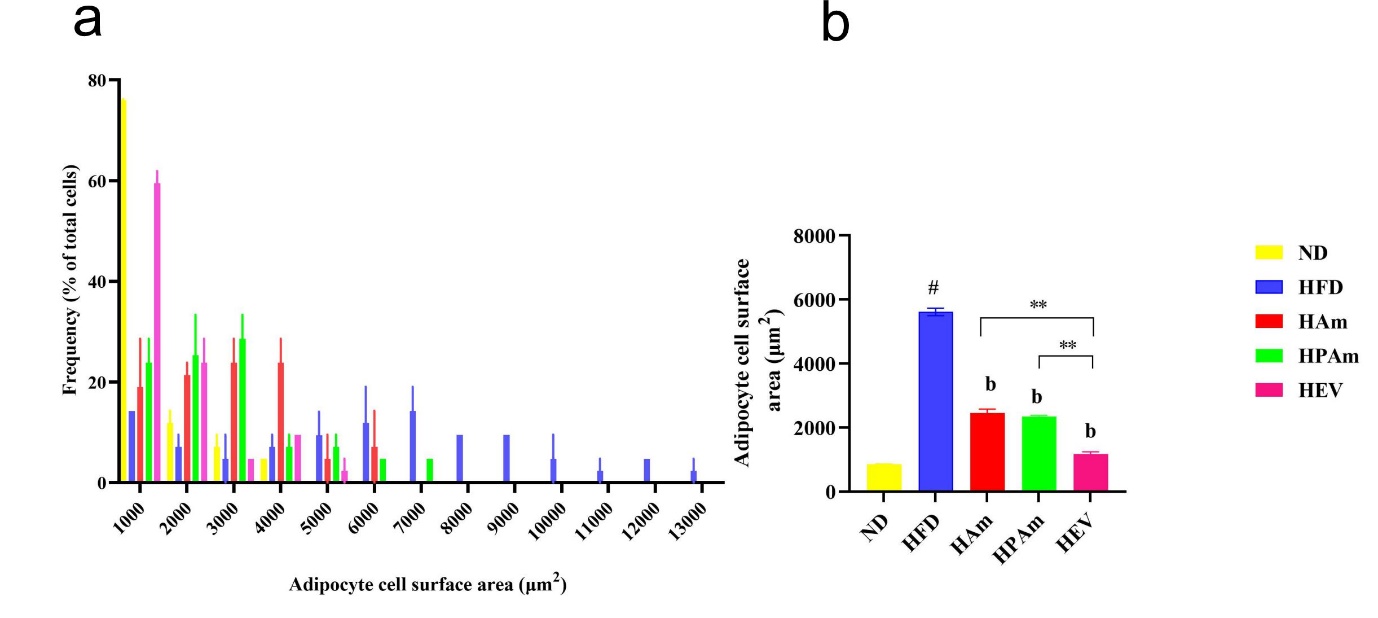


**Fig. S2** The effects of live, pasteurized *A. muciniphila* and its EVs on changes in adipocyte cell surface area. **a** Frequency distribution of adipocyte cell surface. **b** Mean surface area of adipocyte. #, p < 0.05; HFD vs. ND, a, p < 0.05; b, p < 0.01; treatment groups vs. HFD, and *, p < 0.05; **, p < 0.01; among treatment groups were considered statistically significant, respectively. ND: normal diet + PBS, HFD: high fat diet + PBS, HAm: high fat diet + A. muciniphila (109 CFU), HPAm: high fat diet + pasteurized A. muciniphila (109 CFU), and HEV: high fat diet + EVs (10 µg protein).

**Table S1.** List of datasets used in meta-analysis of selected genes in liver of C57BL/6 mice.

| **GEO series ID** | **GEO platform ID** | **No. of ND sample** | **No. of HFD sample** | **Time**  **(week)** | **Submission date** |
| --- | --- | --- | --- | --- | --- |
| **GSE80630** | GPL1261  ([Mouse430_2] Affymetrix Mouse Genome 430 2.0 Array) | 10 | 40 | 4 | 2016 |
| **GSE39549** | GPL6887  (Illumina MouseWG-6 v2.0 expression beadchip) | 3 | 3 | 4,6 | 2014 |
| **GSE63175** | GPL19146  (Agilent-025293 Mouse Whole Genome 4x44k Microarray (Probe Name version))) | 3 | 3 | 6 | 2014 |
| **GSE12409** | GPL6962  (University of Lausanne Mouse 17K (M17K) array version 1) | 6 | 6 | 4 | 2009 |

**Table S2.** Sequence of primers used in qPCR in mice.

| **Primer name** | **Forward primer (5`-3`)** | **Reverse primer (5`-3`)** | **Product size (bp)** |
| --- | --- | --- | --- |
| ***rpl-19*** | TCAGCCACAACATTCTCA | GCACCTCCAACAGTAAGT | 138 |
| ***tlr-2*** | TCCTGCGAACTCCTATCC | CCTGGTGACATTCCAAGAC | 151 |
| ***tlr-4*** | GCCTTTCAGGGAATTAAGCTCC | GATCAACCGATGGACGTGTAAA | 114 |
| ***zo-1*** | GCCGCTAAGAGCACAGCAA | TCCCCACTCTGAAAATGAGGA | 134 |
| ***cldn-1*** | TCTGCCACTTCTCACTTCCA | GCCTATACCCTTGCTCTCTGT | 95 |
| ***cldn-2*** | CAACTGGTGGGCTACATCCTA | CCCTTGGAAAAGCCAACCG | 128 |
| ***ocldn*** | TTGAAAGTCCACCTCCTTACAGA | CCGGATAAAAAGAGTACGCTGG | 129 |
| ***il-10*** | GCACTACCAAAGCCACAAG | AGTAAGAGCAGGCAGCATAG | 85 |
| ***tnf-α*** | AACAACTACTCAGAAACACAAG | GCAGAACTCAGGAATGGA | 130 |
| ***angptl4*** | ACTGTGAGATGACTTCAGATGG | ATTGGCTTCCTCGGTTCC | 174 |
| ***hprt*** | TCAGTCAACGGGGGACATAAA | GGGGCTGTACTGCTTAACCAG | 142 |
| ***ppar-α*** | CACTTGCTCACTACTGTCCTT | GATGCTGGTATCGGCTCAA | 110 |
| ***ppar-γ*** | GGTGCTCCAGAAGATGACAGA | TCAGCGGGTGGGACTTTC | 154 |
| ***tgf-β1*** | AATTCCTGGCGTTACCTT | TGTATTCCGTCTCCTTGG | 116 |
| ***il-6*** | TCCATCCAGTTGCCTTCT | TAAGCCTCCGACTTGTGAA | 137 |
| ***lpl*** | TTCTCCTGATGACGCTGATT | TCACACGGATGGCTTCTC | 218 |

**Table S3.** Sequence of primers used for the gut microbiota analysis.

| **Primer name** | **Forward primer (5`-3`)** | **Reverse primer (5`-3`)** |
| --- | --- | --- |
| **Firmicute** | GGAGYATGTGGTTTAATTCGAAGCA | AGCTGACGACAACCATGCAC |
| **Bacteroidetes** | GTTTAATTCGATGATACGCGAG | TTAASCCGACACCTCACGG |
| **Actinobacteria** | TGTAGCGGTGGAATGCGC | AATTAAGCCACATGCTCCGCT |
| ***Verrucomicrobia*** | TCAKGTCAGTATGGCCCTTAT | CAGTTTTYAGGATTTCCTCCGCC |
| **Fusobacteria** | GATCCAGCAATTCTGTGTG | CGAATTTCACCTCTACACTTG |
| ***Clostridia*** | AAATGACGGTACCTGACTAA | CTTTGAGTTTCATTCTTGCGAA |
| ***γ-Proteobacteria*** | TCGTCAGCTCGTGTYGTGA | CGTAAGGGCCATGATG |
| ***α-Proteobacteria*** | CIAGTGTAGAGGTGAAATT | CCCCGTCAATTCCTTTGAGTT |
| ***ε-Proteobacteria*** | TAGGCTTGACATTGATAGAATC | CTTACGAAGGCAGTCTCCTTA |
| ***Enterobacteriaceae*** | CATTGACGTTACCCGCAGAAGAAGC | CTCTACGAGACTCAAGCTTGC |
| ***Rumminococcaceae*** | GGCGGCYTRCTGGGCTTT | CCAGGTGGATWACTTATTGTGTTAA |
| ***Peptostreptococcus*** | AACTCCGGTGGTATCAGATG | GGGGCTTCTGAGTCAGGTA |
| ***Prevotellaceae*** | AACCCGTTGGGTGTGCC | AGIGCCCAAACCTCCATCTCTCC |
| ***Methanobrevibacter* spp.** | CGATGCGGACTTGGTGTTG | TGTCGCCTCTGGTGAGATGTC |
| ***Bifidobacterium* spp.** | TCGCGTCYGGTGTGAAAG | CCACATCCAGCRTCCAC |
| ***F. prausnitzii*** | GATGGCCTCGCGTCCGATTAG | CCGAAGACCTTCTTCCTCC |
| ***A. muciniphila*** | CAGCACGTGAAGGTGGGGAC | CCTTGCGGTTGGCTTCAGAT |
| ***Lactobacillus* spp.** | AGCAGTAGGGAATCTTCCA | CACCGCTACACATGGAG |
| ***Veillonella* spp.** | ACAACCTGCCCTTCAGA | CGTCCCGATTAACAGAGCTT |
| ***Enterococcus* spp.** | CCCTTATTGTTAGTTGCCATCATT | ACTCGTTGTACTTCCCATTGT |
| ***Alistipes* spp*.*** | TTAGAGATGGGCATGCGTTGT | TGAATCCTCCGTATTACCGCG |
| ***E. coli*** | CATTGACGTTACCCGCAGAAGAAGC | CTCTACGAGACTCAAGCTTGC |
| ***Roseburia* spp.** | TACTGCATTGGAAACTGTCG | CGGCACCGAAGAGCAAT |
| **Universal** | AAACTCAAAKGAATTGACGG | CTCACRRCACGAGCTGAC |

**Additional methods**

**Preparation of *A. muciniphila* EVs**

**Scanning Electron Microscopy (SEM)**

SEM was used to verify the stability and maintenance of the structures and shapes of *A. muciniphila* EVs, during the extraction steps. For this purpose, after washing with 0.1 M PBS and fixing with 2.5% glutaraldehyde solution (at 4°C), the EVs were dehydrated using a graded ethanol series (10 min per step). The dehydrated sample was critically dried with liquid carbon dioxide for 1 hour and covered with gold-palladium by sputter coating. Finally, the EVs were observed by a Field Emission scanning electron microscope (FE-SEM) (HITACHI CS-4160).

**SDS-PAGE**

The extracted EVs corresponding to 10 μg of protein were re-suspended in SDS-PAGE sample buffer (1 M Tris HCl [pH 6.8], 10% SDS, 1% bromophenol blue, glycerol, and β-mercaptoethanol) and heated for 10 min at 95°C. At the end, each sample were loaded and run. The proteins were separated on a 12% SDS-PAGE gel, and protein bands were visualized by a Coomassie brilliant blue.

**Histological** **analysis**

The colon, liver and adipose tissues were immersed in 10% buffered formalin. Then, tissues were dehydrated in ascending graded series (70, 80, 90 and 100 (×2) %) of ethanol. They cleared in xylene (×2) and impregnated and embedded in paraffin. Paraffin blocks were cut using manual rotary microtome at 6 μm thickness and mounted on glass slides. For histological evaluation, the sections were stained with hematoxylin and eosin (H&E). Dino-lite digital lens, Dino Capture 2 software (AnMo 423 Electronics Corp., Taiwan), and light microscope (Olympus SX-21) were used for histopathological analysis and the measurement of frequency distribution of adipocyte cell surface area (µm^2^).
